# A “*Blood Relationship”* Between the Overlooked Minimum Lactate Equivalent and Maximal Lactate Steady State in Trained Runners. Back to the Old Days?

**DOI:** 10.3389/fphys.2018.01034

**Published:** 2018-07-31

**Authors:** Ibai Garcia-Tabar, Esteban M. Gorostiaga

**Affiliations:** Studies, Research and Sports Medicine Center, Government of Navarre, Pamplona, Spain

**Keywords:** lactate threshold, aerobic capacity, Owles' point, oxygen endurance performance limit, aerobic threshold, anaerobic threshold, endurance assessment, submaximal exercise testing

## Abstract

Maximal Lactate Steady State (MLSS) and Lactate Threshold (LT) are physiologically-related and fundamental concepts within the sports and exercise sciences. Literature supporting their relationship, however, is scarce. Among the recognized LTs, we were particularly interested in the disused “Minimum Lactate Equivalent” (LE_min_), first described in the early 1980s. We hypothesized that velocity at LT, conceptually comprehended as in the old days (LE_min_), could predict velocity at MLSS (_V_MLSS) more accurate than some other blood lactate-related thresholds (BL_R_Ts) routinely used nowadays by many sport science practitioners. Thirteen male endurance-trained [_V_MLSS 15.0 ± 1.1 km·h^−1^; maximal oxygen uptake (${\dot{V}O}_{2max}$) 67.6 ± 4.1 ml·kg^−1^·min^−1^] homogeneous (coefficient of variation: ≈7%) runners conducted 1) a submaximal discontinuous incremental running test to determine several BL_R_Ts followed by a maximal ramp incremental running test for ${\dot{V}O}_{2max}\;$determination, and 2) several (4–5) constant velocity running tests to determine _V_MLSS with a precision of 0.20 km·h^−1^. Determined BL_R_Ts include LE_min_ and LE_min_-related LE_min_ plus 1 (LE_min+1mM_) and 1.5 mmol·L^−1^ (LE_min+1.5mM_), along with well-established BL_R_Ts such as conventionally-calculated LT, D_max_ and fixed blood lactate concentration thresholds. LE_min_ did not differ from LT (*P* = 0.71; ES: 0.08) and was 27% lower than MLSS (*P* < 0.001; ES: 3.54). LE_min+1mM_ was not different from MLSS (*P* = 0.47; ES: 0.09). LE_min_ was the best predictor of _V_MLSS (*r* = 0.91; *P* < 0.001; SEE = 0.47 km·h^−1^), followed by LE_min+1mM_ (*r* = 0.86; *P* < 0.001; SEE = 0.58 km·h^−1^) and LE_min+1.5mM_ (*r* = 0.84; *P* < 0.001; SEE = 0.86 km·h^−1^). There was no statistical difference between MLSS and estimated MLSS using LE_min_ prediction formula (*P* = 0.99; ES: 0.001). Mean bias and limits of agreement were 0.00 ± 0.45 km·h^−1^ and ±0.89 km·h^−1^. Additionally, LE_min_, LE_min+1mM_ and LE_min+1.5mM_ were the best predictors of ${\dot{V}O}_{2max}$ (*r* = 0.72–0.79; *P* < 0.001). These results support LE_min_, an objective submaximal overlooked and underused BL_R_T, to be one of the best single MLSS predictors in endurance trained runners. Our study advocates factors controlling LE_min_ to be shared, at least partly, with those controlling MLSS.

## Introduction

The exercise intensity corresponding to the maximal lactate steady state (MLSS) is a consistent physiological phenomenon describing the highest constant velocity or power output that can be maintained over time without continual blood lactate concentration (BLC) accumulation (Beneke, 1995). Nowadays MLSS is considered the gold standard endurance performance marker among the vast majority of sport and exercise science physiologists (Beneke, 1995; Llodio et al., 2016; Messias et al., 2017). MLSS is valuable, and more sensitive than maximal oxygen uptake (${\dot{V}O}_{2max}$), to diagnose endurance performance (Coyle et al., 1988), guide aerobic training (Haverty et al., 1988), evaluate endurance training-induced adaptations (Philp et al., 2008) and predict endurance performance (Haverty et al., 1988; Jones and Doust, 1998). Determination of MLSS is, however, cumbersome and interferes with the athlete's training program since it requires several (3–6) constant workload tests on separate days lengthening aerobic conditioning evaluation to a minimum of 1–3 week period (Heck et al., 1985).

In an attempt to overcome the shortcomings of multiple-day testing, simpler methods have been proposed to estimate MLSS from a single-day test, involving the use of either BLC-based measurements or some other bloodless simple measurements such as the peak workload reached during an incremental maximal test. Numerous studies conducted on competitive athletes have shown that the intensities corresponding to some blood lactate-related thresholds (BLRTs), such as the Onset of Blood Lactate Accumulation (OBLA) (Beneke, 1995; Van Schuylenbergh et al., 2004), Individual Anaerobic Threshold (IAT) (Beneke, 1995), D_max_ (Van Schuylenbergh et al., 2004) or the Lactate Minimum Test (LMT) (Jones and Doust, 1998), predict MLSS with a wide range of correlation magnitudes (*r* = 0.61–0.85). However, these correlation magnitudes are equal, or even lower, than the ones reported in those same studies when the peak workload attained during an incremental maximal test was used as MLSS predictor (*r* = 0.85–0.94).

Before the appearance of the MLSS concept and based on the early works of Barr and Himwich (1923) and Owles (1930) published in the 1920s, several researchers independently found that during graded incremental exercise there is a critical exercise intensity level unique to each individual above which BLC initiates to increase beyond resting values. In the following years this critical workload level, which always occurs at lower intensities than MLSS (Lehmann et al., 1983; Aunola and Rusko, 1988; Faude et al., 2009; Ferguson et al., 2018) and is frequently called “*Lactate Threshold* (LT)” (Jones and Ehrsam, 1982) [although it has also been termed “*Owles' Point*” (Jones and Ehrsam, 1982), “*Oxygen Endurance Performance Limit*” (Hollmann, 1985), “*Aerobic Threshold*” (Kindermann et al., 1978) or “*Anaerobic Threshold*” (Wasserman et al., 1973)], was widely considered as the standard criterion measure to determine aerobic capacity (Weltman et al., 1987; Mezzani et al., 2012), predict endurance performance (Yoshida et al., 1990), and design endurance exercise training programs (Weltman et al., 1990); turning LT into a pivotal concept within the sports medicine and exercise sciences. Notwithstanding, there are still some relevant methodological limitations on the accurate and rigorous determination of LT, mainly when (a) it is determined by simple visual inspection of BLC-data plotted against workload due to the subjectivity of the analysis and poor inter-viewer and inter-method agreement (Yeh et al., 1983), (b) the initial workload and subsequent initial workload increments are not low enough to allow a preliminary BLC-baseline phase on the BLC kinetics during the graded exercise (Hollmann, 1985), and (c) the BLC-data-point interval is too large to detect LT with a suitable sensitivity (Hollmann, 1985). Beyond a shadow of a doubt, objective methodological approaches and appropriate rigorous protocols are needed to overcome these limitations (Brooks, 1985).

Despite MLSS and LT being physiologically different, but probably related, fundamental concepts within the sports and exercise sciences (Ferguson et al., 2018), literature concerning their relationship is scarce. As far as the authors are aware, whether the velocity at LT (_V_LT) obtained during an incremental exercise test predicts the velocity at MLSS (_V_MLSS) in endurance trained runners has not been fully explored, and deserves further attention. We hypothesized that _V_LT, conceptually comprehended as in the old days (Owles, 1930), could predict _V_MLSS more accurate than some others BL_R_Ts used nowadays by many authors and other sport science practitioners. Accordingly, the primary purpose of this study was to determine the applicability of the classical gold standard _v_LT, calculated objectively and in a standardized manner, to predict _V_MLSS in comparison with some other more commonly used parameters of BLC changes during incremental exercise in a homogeneous group of endurance trained runners. Among the recognized BL_R_Ts (Faude et al., 2009) we were particularly interested in the “Minimum Lactate Equivalent” (LE_min_), initially described by German authors in the early 1980s (Berg et al., 1980; Lehmann et al., 1983). LE_min_ is the minimum value of the BLC/workload vs. workload curve fitting during an incremental exercises test. Using an appropriate protocol with adequate opening and incremental workloads, the incremental test produces an idiosyncratic “U-shaped” curve fitting profile allowing mathematical impartial location of the transition at _V_LT with a very fine resolution. This seldom used method (LE_min_) should not be confused with the much more popular “Lactate Minimum Test” (LMT), which was originally described by Tegtbur et al. (1993) and uses a preliminary relatively high level of exertion phase (hyperlactatemia phase) to set-up the mentioned “U-shaped” curve fitting profile hampering heart rate (HR) data interpretation, and therefore, its on-field application.

A secondary purpose of this study was to determine the extent to which some variables not requiring blood sampling, such as ${\dot{V}O}_{2max}$, peak treadmill velocity (PTV) or the velocity corresponding to the 90% of maximal heart rate (V90) (Garcia-Tabar et al., 2015b), are of potential interest to estimate _V_MLSS. To the best of our knowledge literature concerning _V_MLSS prediction from such variables in well-trained endurance runners is limited. Assessment and monitoring of aerobic capacity in this kind of athletes is of paramount importance (Halson, 2014), and consequently, this study has the potential to contribute with noteworthy scientific-based practical endurance performance implications.

## Materials and methods

### Subjects

Fifteen male trained middle- and long-distance runners were recruited from regional athletic clubs. Runners were required to meet the following inclusion criteria: (1) being male runners aged between 18 and 40; (2) having a _V_MLSS >13 km·h^−1^, and (3) a training routine of ≥3 aerobic running training sessions per week. Exclusions criteria were: (1) being taking any medication/supplementation that could affect BLC or HR values and (2) having any known cardiovascular, respiratory or circulatory dysfunction. One runner withdrew from the study due to personal reasons and another runner did not meet the inclusion criteria. Thirteen runners completed the study. Mean (±SD) age, height, body mass and percentage of body fat of the thirteen participants were 28 ± 7 y, 1.76 ± 0.05 m, 68.8 ± 6.8 kg and 8.8 ± 3.1%, respectively. Runners competed in races ranging from 800-m to half-marathon.

The study was conducted according to the guidelines laid down in the Declaration of Helsinki and all procedures were approved by the local Institutional Review Committee of the Instituto Navarro del Deporte y Jueventud (Government of Navarre, Spain). Inclusion and exclusion criteria, experimental rationale, testing procedures and associated risks and benefits of participation were fully explained to participants and their coaches by an oral presentation. Prior to any testing, participants acknowledged voluntary participation through written informed consent.

### Study design

A predictive cross-sectional study was conducted to determine _V_MLSS from a single-session submaximal discontinuous incremental running test (SD-IRT). Participants conducted 7–8 laboratory testing sessions. (1) Heath screening session: a maximal ramp incremental cycling test to discard any cardiovascular anomaly (12-lead electrocardiogram, GE Healthcare, CASE Marquette, Germany). (2) Familiarization session: a SD-IRT to accustom to the testing treadmill running protocol. This session was also utilized for anthropometric evaluation. (3) BL_R_Ts and ${\dot{V}O}_{2max}$ testing session: the SD-IRT previously used in the familiarization session to determine BL_R_Ts, followed by a maximal ramp incremental running test (MR-IRT) to determine ${\dot{V}O}_{2max}$. (4) _V_MLSS testing: 4–5 constant velocity running tests (CVRTs) for _V_MLSS determination.

### Testing procedures

Participants were required to complete the study within 6 weeks. Testing sessions were performed at the same time of the day to lessen circadian variability, were preceded by 2 days of rest or very light exercise [<90 min at <70% maximal HR (HR_max_)] and were separated from the last competitive race by ≥4 days to allow restoration of muscle glycogen. Runners arrived to each testing session in a rested and fully hydrated state, 2 h postprandial, having abstained from caffeinated and alcoholic beverages during the day. Subjects were asked to replicate diet and exercise regimens the 2 days preceding each testing session to limit fluctuations of initial glycogen concentration between trials (Van Schuylenbergh et al., 2004; Philp et al., 2008). Participants recorded their exercise training and diet throughout the experimental phase of the study on training and diet logs designed and provided by the authors. These detailed exercise and diet logs served to confirm fulfillment of diet and exercise instructions given for the 2 days preceding each testing session, and verified that only minor changes in training and aerobic conditioning occurred during the study (Farrell et al., 1979). Participants wore the same running trainers on each experimental day. Testing took place during May-June, i.e., beginning of the outdoor competitive season. All procedures were conducted on the same running ergometer (Kuntaväline, Hyper Treadmill 2040, Finland) with the gradient set at 1%, under temperature (22.3 ± 1.4°C), humidity (33 ± 4%) and luminosity controlled laboratory conditions.

#### BL_R_Ts and ${\dot{V}O}_{2max}$ testing

Athletes performed a SD-IRT for BL_R_Ts determination, followed by a MR-IRT to determine their ${\dot{V}O}_{2max}$. The submaximal trial began at 7 km·h^−1^. Speed was increased by 1 km·h^−1^ every 2-min, with 1-min intervals between stages until a BLC ≥3 mmol·L^−1^ was observed. On the basis that 1- to 4-min stage duration protocols do not notably affect BL_R_Ts detection (Yoshida, 1984), 2-min duration stages were chosen not to unnecessary lengthen the SD-IRT according to previous LE_min_ detection protocols (Berg et al., 1980, 1990; Lehmann et al., 1983; Aunola and Rusko, 1988). Immediately after each stage, capillary blood samples for BLC measurements were obtained. After a 10-min rest, subjects began the MR-IRT. Initial speed was 10 km·h^−1^ and was increased by 1 km·h^−1^ every min until volitional exhaustion. Volunteers were vigorously encouraged to complete exhaustion. Post-exercise capillary blood samples after 3 min of passive recovery were obtained for peak BLC (BLC_peak_) determination. HR during both trials (Polar Electro Oy, RS800CX, Finland) and metabolic data during the MR-IRT (Vista Mini-CPX, Vacu-Med, Silver Edition 17670, Ventura, CA, USA) were monitored and averaged over 30-s. PTV, HR_max_ (Garcia-Tabar et al., 2017) and ${\dot{V}O}_{2max}$ (Garcia-Tabar et al., 2015a) were determined following procedures previously described.

#### _V_MLSS testing

On subsequent laboratory visits, runners completed 4–5 CVRTs. Each CVRT consisted of 30-min running at the selected speeds with 1-min interruptions every 10-min for blood sampling (i.e., 32-min duration CVRTs). Capillary blood samples were obtained at rest, and at min 10, 21 and 32 of exercise. An increase in BLC <1.0 mmol·L^−1^ during the last 20 min of exercise (i.e., between the 10th and the 32nd min of the CVRT) was defined as the criterion for BLC to be considered at a steady state (Beneke, 1995). _V_MLSS was defined as the highest running velocity meeting this stability criterion. Running velocity of the first CVRT corresponded to approximately 80% of the PTV achieved during the maximal trial. Depending on the BLC stability of this first CVRT, the velocity was increased or decreased in the following CVRTs. If during the first CVRT a steady state or decrease in BLC was found, the velocity for the next CVRT was increased by 0.4 km·h^−1^. Conversely, if an increase in BLC superior to the stability criterion was observed, running velocity for the next CVRT was decreased by 0.4 km·h^−1^. This process of increasing or decreasing running velocity by 0.4 km·h^−1^, and later by 0.2 km·h^−1^, was further repeated in subsequent tests until _V_MLSS was determined with a precision of 0.2 km·h^−1^. HR was monitored and averaged as abovementioned.

### Blood sampling and blood lactate concentration (BLC) determination

A hyperemic earlobe was cleaned and dried before puncturing by a lancet device to aspirate a 5 μL whole blood sample into an enzyme-coated electrode test strip. BLC was determined via amperometric measurement using a portable analyzer (Arkray KDK Corporation, Lactate Pro LT-1710, Shiga, Japan) calibrated before every test. Manufacturers report coefficients of variation (CVs) of 3.2 and 2.6% for lactate standards of 2 and 11 mmol·L^−1^, respectively.

### Determination of blood lactate-related thresholds (BL_R_Ts)

**Figure 1:**
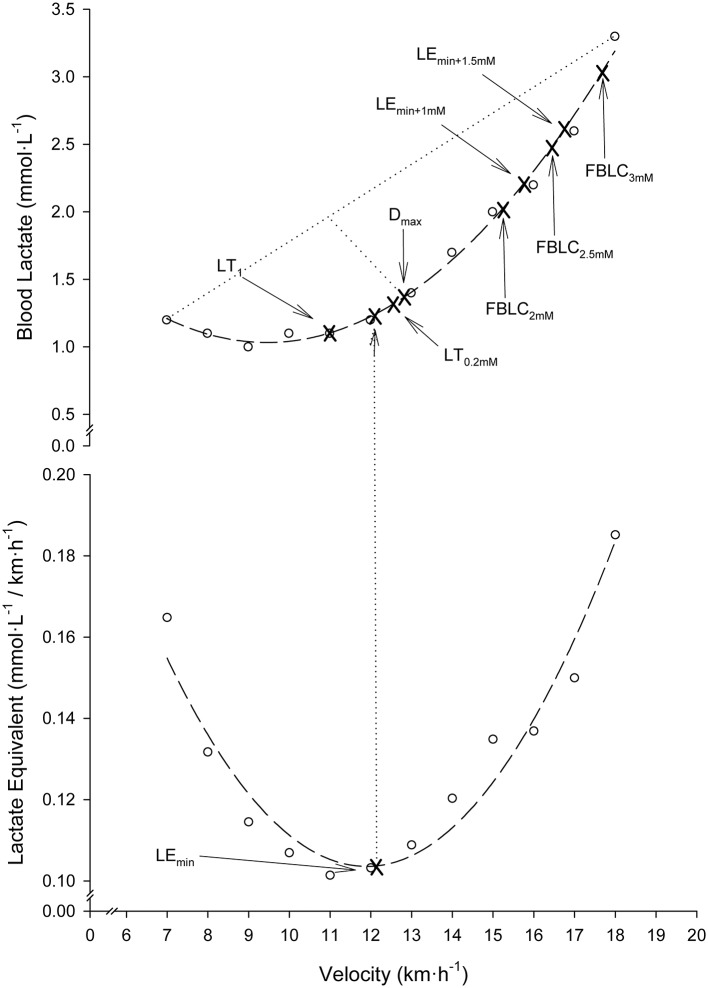
Illustration of blood lactate-related thresholds (BL_R_Ts) determination in a representative participant. Dashed lines: second-order polynomial curve fits. Dotted lines: the greatest perpendicular distance from the third-order polynomial BLC-velocity curve fit to the generated straight line by the two end data-points of this curve. Note that for clearness of figure presentation, D_max_ determination is illustrated together with the rest of BL_R_Ts on a second-order polynomial curve fit, although actually it was determined on third-order curvilinear fits as originally described (Cheng et al., 1992).

Nine different BL_R_Ts were determined. LT_0.2mM_ and LT_1_. LT_0.2mM_ was defined as the stage prior to a ≥0.2 mmol·L^−1^ BLC elevation above baseline values (Stratton et al., 2009). To overcome the error associated with the analyzer (Weltman et al., 1987), the highest stage above which BLC increased by ≥0.1 mmol·L^−1^ in the following stage and ≥0.2 mmol·L^−1^ in the subsequent stage was also chosen as a threshold and named LT_1_. LE_min_, LE_min+1mM_ and LE_min+1.5mM_. The velocity corresponding to the Minimum Lactate Equivalent (_V_LE_min_) (Berg et al., 1990) was considered the minimum value of the quotient BLC/velocity in the individual BLC/velocity vs. velocity second-order polynomial curves. Velocity associated with the Minimum Lactate Equivalent plus 1 (_V_LE_min+1mM_) and 1.5 mmol·L^−1^ (_V_LE_min+1.5mM_) were defined as the running velocities at 1.0 and 1.5 mmol·L^−1^ above _V_LE_min_ in the individual BLC vs. velocity second-order polynomial curves, respectively. *D*_*max*_. Velocity at D_max_ was considered the maximum perpendicular distance from the straight line between the first and final BLC data-points to the third-order polynomial curve describing the BLC kinetics during the SD-IRT (Cheng et al., 1992). Fixed blood lactate concentration (FBLC) thresholds. Velocities at FBLC thresholds of 2 (FBLC_2mM_), 2.5 (FBLC_2.5mM_) and 3 mmol·L^−1^ (FBLC_3mM_) commonly use in real practice (Seiler, 2010; Garcia-Tabar et al., 2017) were determined from the individual BLC vs. velocity second-order polynomial curves. Determination of BL_R_Ts is illustrated in Figure 1.

Velocities at the BL_R_Ts were determined using MATLAB R2015a (The MathWorks Inc., Natick, MA, USA). Coefficients of determination (*R*^2^) of the individual second- and third-order BLC vs. velocity and second-order BLC/velocity quotient vs. velocity polynomial curves were all >0.90. Velocities at BL_R_Ts (Weltman et al., 1990), as well as _V_MLSS (Hauser et al., 2013), frequently show test-retest intraclass correlation coefficients >0.94, and CVs ≤3%. HR values at the BL_R_Ts were computed from the individual HR vs. velocity linear regression equations (*r* > 0.98; *P* < 0.001). V90 was also calculated from the individual linear HR vs. velocity regressions obtained during the SD-IRTs (Garcia-Tabar et al., 2017).

### Statistics

Standard statistical methods were used for the calculation of means, standard deviations (SD), standard errors of the estimates (SEE) and confidence intervals (CI). Data were analyzed using parametric statistics following confirmation of normality (Kolmogorov–Smirnov test), homoscedasticity (Levene's test), and when appropriate sphericity (Mauchly's test). The Greenhouse-Geisser correction factor to reduce the risk of type I error was applied where sphericity assumptions were violated. Student's paired *t-*tests were used to evaluate differences between each BL_R_T with MLSS. The magnitudes of the differences were assessed using 90% CI and Hedges' *g* effect sizes (ES) (Hedges, 1981). Differences were considered non-substantial if the 90% CIs overlapped zero. ES values of 0.2, 0.5, and >0.8 were considered to represent small, moderate, and large differences, respectively. Differences in BLC and HR along the CVRTs were identified by one-way repeated measures ANOVA with Bonferroni correction for multiple comparisons. Two-factorial ANOVA with the Scheffé post-hoc test was used to identify differences in BLC and HR between the CVRTs at _V_MLSS and at 0.2 km·h^−1^ above _V_MLSS (_V_MLSS_+0.2_). Linear regression analyses with Pearson's correlation coefficients (*r*) were performed to determine the relationships between the variables of interest. When pertinent, slopes of regression lines were compared using analysis of covariance (ANCOVA). Agreement with the reference method (_V_MLSS) was assessed by mean bias and limits of agreement (LOAs) (Krouwer, 2008). Post-hoc power calculation for the linear regressions, assuming type I error of 0.05, indicated a power >99%. Analyses were performed using IMB SPSS Statistics 22 (IBM Corporation, NY, USA). Significance was set at *P* < 0.05 for the analyses that did not require post-hoc adjustment. Descriptive statistics are reported as means (±SD).

## Results

### BL_R_Ts and ${\dot{V}O}_{2max}$ testing

**Figure 2:**
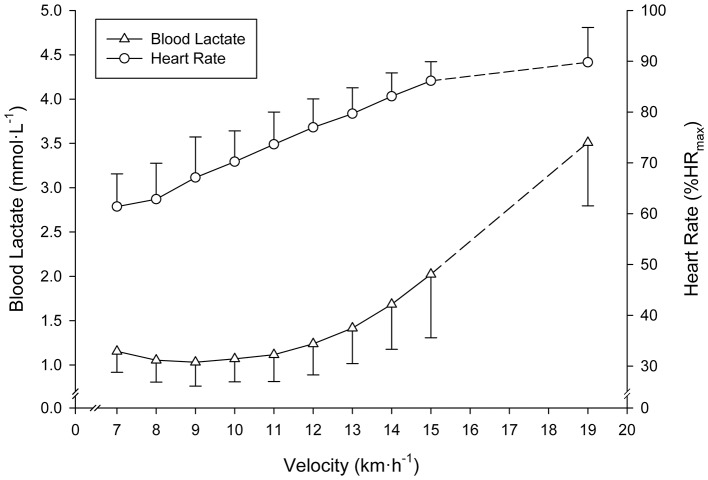
Mean (SD) blood lactate and heart rate responses to the submaximal discontinuous incremental running exercise test. All subjects terminated the 15 km·h^−1^ exercise stage. Mean (SD) values at completion of the test of subjects achieving ≥16 km·h^−1^ are indicated by dashed lines.

The SD-IRT lasted 32:00 ± 4:24 min:s. Runners achieved a treadmill velocity of 17.0 ± 1.5 km·h^−1^ (range 15.0–19.0). BLC and %HR_max_ at completion of the SD-IRT were 3.4 ± 0.6 mmol·L^−1^ (range 3.0–5.4) and 92 ± 2% (range 87–93), respectively. Figure 2 depicts BLC and %HR_max_ pattern responses to the SD-IRT. Descriptive characteristics of the BL_R_Ts are depicted in Table 1. BLC resting values prior to the beginning of the MR-IRT were 1.1 ± 0.2 mmol·L^−1^ (range 0.8–1.7). Table 2 elucidated the maximal nature of the MR-IRT.

**Table 1:** Descriptive features of the determined blood lactate-related thresholds and maximal lactate steady state (MLSS) (*n* = 13).

	**km**·**h**^**−1**^		**%MLSS**_**V**_		**%PTV**		**%HR**_**max**_	
	**Mean ± *SD***	**Range**	**Mean ± *SD***	**Range**	**Mean ± *SD***	**Range**	**Mean ± *SD***	**Range**
LE_min_	11.6 ± 0.8^**^	10.5–12.6	77 ± 2^**^	74–80	58 ± 3^**^	55–64	75 ± 5^**^	63–80
LT_1_	11.7 ± 1.7^**^	9.0–14.0	78 ± 7^**^	64–88	59 ± 7^**^	47–70	76 ± 4^**^	66–83
LT_0.2mM_	12.5 ± 1.4^**^	10.0–15.0	84 ± 6^**^	73–94	63 ± 5^**^	52–70	79 ± 3^**^	70–83
D_max_	13.2 ± 1.2^**^	11.5–14.6	87 ± 4^**^	81–95	66 ± 4^**^	59–73	81 ± 2^**^	77–85
FBLC_2mM_	14.8 ± 1.5	12.8–17.0	99 ± 7	84–107	75 ± 5	67–82	86 ± 3^*^	80–90
MLSS	15.0 ± 1.1	13.3–16.5	100 ± *N*/*A*	N/A	76 ± 4	69–82	91 ± 4	83–95
LE_min+1mM_	15.1 ± 1.2	13.7–16.8	101 ± 4	95–106	76 ± 4	70–85	86 ± 3^*^	80–90
FBLC_2.5mM_	15.9 ± 1.5^*^	14.0–18.1	106 ± 6^*^	94–114	80 ± 5^*^	73–88	90 ± 3	84–94
LE_min+1.5mM_	15.9 ± 1.4^*^	13.0–18.1	106 ± 6^*^	94–113	81 ± 5^*^	70–89	90 ± 3	86–93
FBLC_3mM_	16.8 ± 1.5^**^	15.0–19.1	112 ± 6^**^	102–120	85 ± 5^**^	78–93	92 ± 3	87–97

*LE_min_, minimum lactate equivalent; LT_1_, the highest stage above which blood lactate concentration increased by ≥0.1 mmol·L^−1^ in the following stage and ≥0.2 mmol·L^−1^ in the subsequent stage; LT_0.2mM_, the stage prior to a ≥0.2 mmol·L^−1^ blood lactate concentration elevation above baseline values; D_max_, Maximal-Deviation method; FBLC_2mM_, fixed blood lactate concentration (FBLC) threshold of 2 mmol·L^−1^; LE_min+1mM_, LE_min_ plus 1 mmol·L^−1^; FBLC_2.5mM_, FBLC threshold of 2.5 mmol·L^−1^; LE_min+1.5mM_, LE_min_ plus 1.5 mmol·L^−1^; FBLC_3mM_, FBLC threshold of 3 mmol·L^−1^*.

* *Significantly different from MLSS (P < 0.01)*.

** *Significantly different from MLSS (P < 0.001)*.

**Table 2:** Maximal values attained during the maximal ramp incremental running test (*n* = 13).

	**Mean ± SD**	**Range**
Test duration (min:s)	12:36 ± 00 : 54	11:18–14:12
PTV (km·h^−1^)	19.8 ± 0.9	18.6–21.4
$\dot{V}{\text{O}}_{\text{max}}$(ml·kg^−1^·min^−1^)	67.6 ± 4.1	61.8–73.7
$\dot{V}{\text{E}}_{\text{max}}$ (L·min^−1^)	128 ± 12	104–144
HR_max_ (b·min^−1^)	184 ± 9	167–199
HR_max_ (% age predicted HR_max_)	95 ± 5	88–101
RER_max_	1.18 ± 0.05	1.09–1.26
BLC_peak_ (mmol·L^−1^)	7.6 ± 2.0	5.6–11.8

*PTV, peak treadmill velocity; ${\dot{V}O}_{\max}$, maximal oxygen uptake; ${\dot{V}E}_{\max}$, maximal minute ventilation; HR_max_, maximal heart rate; RER_max_, maximal respiratory exchange ratio; BLC_peak_, peak blood lactate concentration*.

### _V_MLSS testing

**Figure 3:**
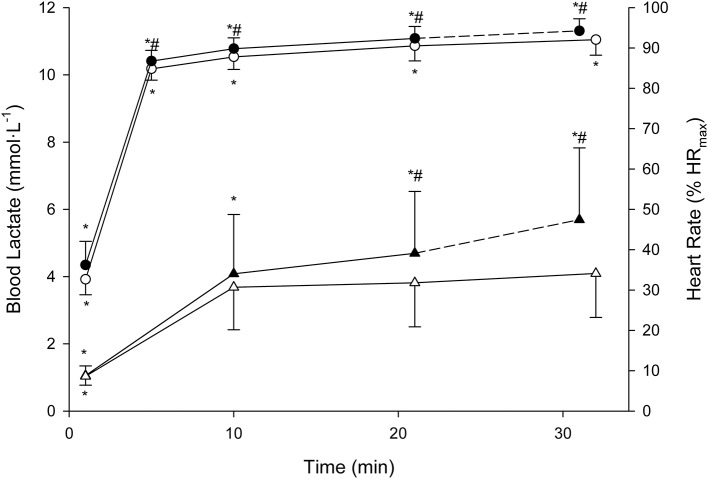
Mean (SD) blood lactate (triangles) and heart rate (circles) responses to the constant running velocities tests (CVRTs) at the maximal lactate steady state velocity (open symbols) and at 0.2 km·h^−1^ faster velocity (filled symbols). * Significantly different from the rest of the time-points within the same CVRT (*P* < 0.0125). ^#^Significantly higher in comparison with the corresponding time-points at the maximal lactate steady state velocity CVRT (*P* < 0.0125).

Descriptive features of the MLSS are displayed along with the BL_R_Ts (Table 1). Velocity at LE_min_ did not differ from that at LT_1_ (*P* = 0.71; 90% CI: −0.74 to 0.47; ES: 0.08) and was 27% lower than _V_MLSS (*P* < 0.001; 90% CI: −3.80 to −2.83; ES: 3.54). Velocities at FBLC_2mM_ (*P* = 0.50; 90% CI: −0.69 to 0.30; ES: 0.15) and LE_min+1mM_ (*P* = 0.47; 90% CI: −0.17 to 0.42; ES: 0.09) were not different from _V_MLSS. V90 (16.1 ± 1.2 km·h^−1^, range 13.9–18.1) was 1.1 km·h^−1^ (7%) higher than _V_MLSS (*P* < 0.001; 90% CI: 0.77–1.53; ES: 0.96), and not different from velocity at FBLC_2.5mM_ (*P* = 0.619; 90% CI: −0.27 to 0.49; ES: 0.09) and _V_LE_min+1.5mM_ (*P* = 0.543; 90% CI: −0.42 to 0.87; ES: 0.15). HR associated with _V_MLSS during the SD-IRT was 86 ± 5% HR_max_, and was not different from %HR_max_ at FBLC_2mM_ (*P* = 0.93; 90% CI: −2.99 to 3.29; ES: 0.01), LE_min+1mM_ (*P* = 0.92; 90% CI: −2.56 to 2.86; ES: 0.04) and FBLC_2.5mM_ (*P* = 0.08; 90% CI: 0.25–6.55; ES: 0.78). BLC and %HR_max_ responses to the CVRTs performed at _V_MLSS and at _V_MLSS_+0.2_ are illustrated in Figure 3. One runner exhausted at min 29 of the CVRT at _V_MLSS_+0.2_, and did not terminate the trial. BLC during the CVRT at _V_MLSS_+0.2_ increased >1 mmol·L^−1^ from the min 10 to the end of the trial (1.6 ± 0.7 mmol·L^−1^; *P* < 0.001; 90% CI: 1.26–1.95; ES: 0.82). During the CVRT at _V_MLSS, BLC from the 10th min to the end of the exercise increased significantly (0.4 ± 0.4 mmol·L^−1^; *P* = 0.02; 90% CI: 0.20–0.63; ES: 0.31), but the increment was <1 mmol·L^−1^ in every single case. This BLC stability criterion was obtained at ≈3.9 ± 1.3 mmol·L^−1^ (range ≈2.1–6.2). HR increased (*P* < 0.01) over the course of both _V_MLSS and _V_MLSS_+0.2_ CVRTs. During the _V_MLSS CVRT, absolute HR increased 7 ± 4 b·min^−1^ (*P* < 0.001; 90% CI: 5.0–9.0; ES: 0.70) from min 10 to the end of the test. HR (%HR_max_) at min 5, 10, 21 and 32 of the _V_MLSS CVRT were 85 ± 3, 88 ± 3, 91 ± 4 and 92 ± 4%, respectively.

### Correlations and agreement between the measured performance variables

**Figure 4:**
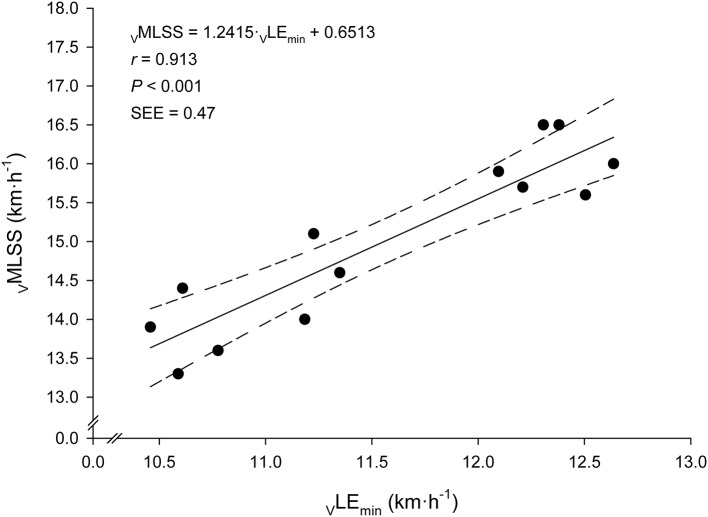
Linear relationship between the velocity at the Minimum Lactate Equivalent (_V_LE_min_) and the velocity at the Maximal Lactate Steady State (_V_MLSS). Solid line: linear regression. Dashed lines: 95% confidence intervals.

Every bloody and bloodless measured endurance performance variables correlated significantly with _V_MLSS (Table 3). _V_LE_min_ was the best predictor of _V_MLSS (Figure 4), followed by _V_LE_min+1mM_ (*r* = 0.86; *P* < 0.001; SEE = 0.58; 95% CI: 0.50–1.13) and _V_LE_min+1.5mM_ (*r* = 0.84; *P* < 0.001; SEE = 0.86; 95% CI: 0.58–1.57). There was no statistical difference between _V_MLSS and estimated _V_MLSS using the formula exposed in Figure 4 (*P* = 0.99; 90% CI: −0.22 to 0.22; ES: 0.001). Mean bias and LOAs were 0.00 ± 0.45 km·h^−1^ and ±0.89 km·h^−1^, respectively, indicating that prediction of _V_MLSS from _V_LE_min_ could be biased up to 5.9% above or below actual _V_MLSS. _V_LE_min+1mM_ did not differ from _V_MLSS (*P* = 0.47; 90% CI: −0.17 to 0.42; ES: 0.09). Mean difference was −0.12±0.6 km·h^−1^ and LOAs were ±1.18 km·h^−1^ (±7.8%).

**Table 3:** Pearson's correlation magnitudes between the selected endurance performance variables (*n* = 13).

	**LE_min_**	**LE_min + 1 mM_**	**LE_min + 1.5 mM_**	**LT_1_**	**D_max_**	**FBLC_3 mM_**	**V90**	**FBLC_2.5 mM_**	**PTV**	**FBLC_2 mM_**	**LT_0.2 mM_**	**${\dot{V}O}_{2\text{max}}$**	**MLSS**
LE_min_		0.947^***^	0.928^***^	0.750^**^	0.756^**^	0.813^***^	0.746^**^	0.778^**^	0.718^**^	0.692^**^	0.674^**^	0.724^**^	0.912^***^
LE_min+1mM_			0.900^***^	0.684^**^	0.719^**^	0.817^***^	0.783^**^	0.768^**^	0.674^*^	0.665^*^	0.713^**^	0.720^**^	0.863^***^
LE_min+1.5mM_				0.703^**^	0.779^**^	0.836^***^	0.596^*^	0.793^***^	0.771^**^	0.706^**^	0.641^*^	0.790^***^	0.839^***^
LT_1_					0.821^***^	0.910^***^	0.860^***^	0.924^***^	0.770^**^	0.920^***^	0.744^**^	0.528	0.836^***^
D_max_						0.895^***^	0.891^***^	0.909^***^	0.743^**^	0.902^***^	0.750^**^	0.374	0.827^***^
FBLC_3mM_							0.850^***^	0.995^***^	0.793^***^	0.967^***^	0.773^**^	0.582^*^	0.804^***^
V90								0.872^***^	0.669^*^	0.889^***^	0.864^***^	0.360	0.799^**^
FBLC_2.5mM_									0.793^***^	0.987^***^	0.773^**^	0.526	0.792^***^
PTV										0.770^**^	0.777^**^	0.703^**^	0.760^**^
FBLC_2mM_											0.745^**^	0.422	0.734^**^
LT_0.2mM_												0.544	0.716^**^
$\dot{V}O_{2\text{max}}$													0.597^*^

LE_min_, minimum lactate equivalent; LE_min+1mM_, LE_min_ plus 1 mmol·L^−1^; LE_min+1.5mM_, LE_min_ plus 1.5 mmol·L^−1^; LT_1_, the highest stage above which blood lactate concentration increased by ≥0.1 mmol·L^−1^ in the following stage and ≥0.2 mmol·L^−1^ in the subsequent stage; D_max_, Maximal-Deviation method; FBLC_3mM_, fixed blood lactate concentration (FBLC) threshold of 3 mmol·L^−1^; V90, velocity corresponding to 90% of maximal heart rate; FBLC_2.5mM_, FBLC threshold of 2.5 mmol·L^−1^; PTV, peak treadmill velocity; FBLC_2mM_, FBLC threshold of 2 mmol·L^−1^; LT_0.2mM_, the stage prior to a ≥0.2 mmol·L^−1^ blood lactate concentration elevation above baseline values; ${\dot{V}O}_{\max}$, maximal oxygen uptake.

* *P < 0.05*,

** *P < 0.01*,

*** *P < 0.001*.

**Figure 5:**
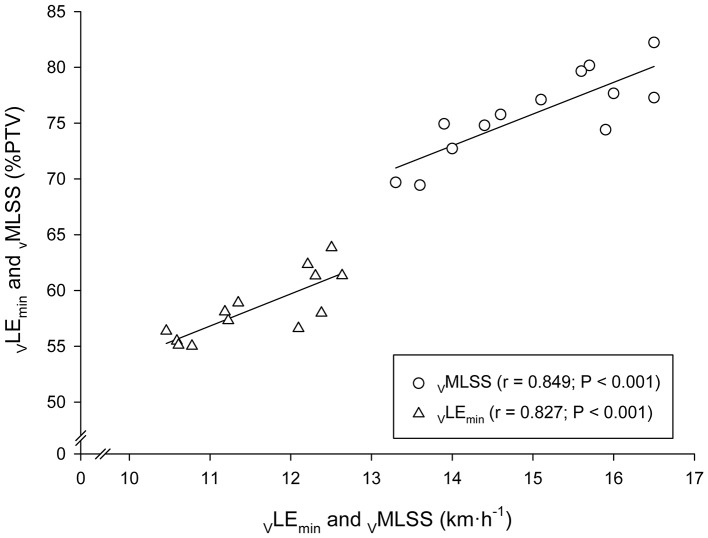
Linear regressions between the velocities at the Minimum Lactate Equivalent (_V_LE_min_) and Maximal Lactate Steady State (_V_MLSS) in absolute values (km·h^−1^) with their respective velocities relative to peak treadmill velocity (PTV).

**Figure 6:**
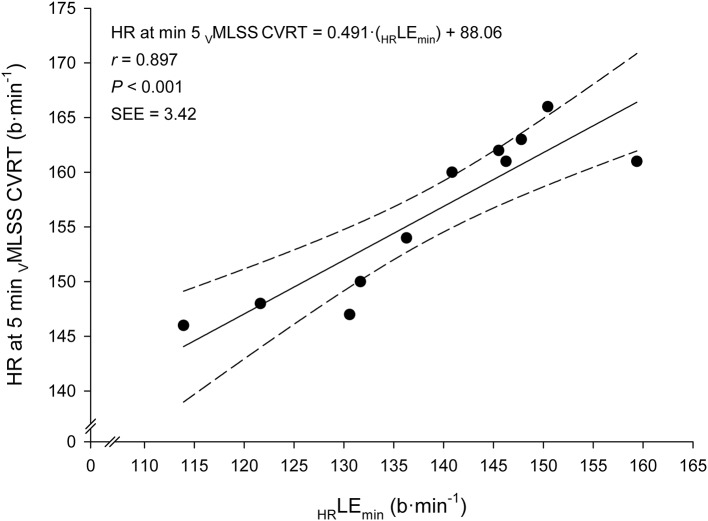
Linear regression between heart rate (HR) at the Lactate Minimum Equivalent (_HR_LE_min_) and HR at min 5 of the constant velocity running test (CVRT) performed at the velocity of the maximal lactate steady state (_V_MLSS).

Very large associations between _V_LE_min_ and _V_MLSS in absolute values (km·h^−1^) with their respective velocities in relative values (%PTV) were observed (Figure 5). According to the ANCOVA results, the slopes of these both regression lines were not different (*P* > 0.05). Very large associations were also found between HR at LE_min_ (_HR_LE_min_) and HR throughout the _V_MLSS CVRT. These correlation magnitudes were *r* = 0.90 (Figure 6), *r* = 0.85 (*P* < 0.001; SEE = 4.9; 95% CI: 0.30–0.83), *r* = 0.79 (*P* = 0.004; SEE = 6.6; 95% CI: 0.25–0.96) and *r* = 0.74 (*P* = 0.009; SEE = 7.9; 95% CI: 0.20–1.06) for HR at min 5, 10, 21, and 32 of the _V_MLSS CVRT, respectively. Due to some technical problems with the HR monitors, HR linear regressions are based upon 11 data-points.

## Discussion

The major finding of this study was that _V_LE_min_ was the strongest predictor of _V_MLSS, followed by _V_LE_min+1mM_, _V_LE_min+1.5mM_, LT_1_ and the rest of the predictor variables (Table 3). These findings are in line with previous research showing that BL_R_Ts, such as OBLA (Beneke, 1995; Van Schuylenbergh et al., 2004; Denadai et al., 2005; Vobejda et al., 2006; Figueira et al., 2008; Grossl et al., 2012), IAT (Beneke, 1995), D_max_ (Van Schuylenbergh et al., 2004), LT (Philp et al., 2008), or other BL_R_Ts (Grossl et al., 2012) obtained during an incremental single-test are significant determinants of MLSS. The high sustained variance by _V_LE_min_ in _V_MLSS prediction in this study (83%, Figure 4) is among the highest reported in the literature (50–88%). Homogeneity of the sample, specificity and characteristics of the test protocol, precision and stability criterion in MLSS determination, as well as the exact variables derived from the incremental test chosen for BL_R_Ts determination are potential factors affecting correlation magnitude differences among studies. For instance, endurance trained runners in the present study were relatively homogeneous in terms of _V_MLSS (CV ≈7%), and determination of their MLSS was very accurate (±0.2 km·h^−1^; ±1.3% mean _V_MLSS). In contrast, study samples in the above-cited publications were more heterogeneous (CVs 7–16%) and precision in MLSS determination was much lower (7–15%), which are factors that can bias comparisons between studies. Concerning the aforementioned studies carried out in runners, LT (Philp et al., 2008) and OBLA (Vobejda et al., 2006) highly correlated with _V_MLSS, accounting for 72 and 81% of the variance, respectively. However, accuracy in _V_MLSS determination (3–4% mean _V_MLSS) was lower than in our study and the study samples composed of male and female runners were heterogeneous (_V_MLSS CVs 12–16%). It is well established that heterogeneity of the samples causes overestimation of correlation magnitudes; the greater the range or the heterogeneity of a group, the greater the magnitude of the correlation coefficient.

With regard to prediction accuracy, it is worth mentioning the relatively low SEE (0.47 km·h^−1^; 3.1% mean _V_MLSS) in _V_MLSS prediction from _V_LE_min_ found in this study (Figure 4). This SEE is lower than the accuracy in MLSS identification commonly utilized (as discussed in the previous paragraph) and compares favorably with other studies predicting MLSS from the intensity associated with OBLA, where SEE values of ≈5.5% (Vobejda et al., 2006; Figueira et al., 2008) and 20.7% (Figueira et al., 2008) of mean MLSS were reported for running and cycling exercise modes, respectively. The Bland-Altman's LOAs (±0.89 km·h^−1^; i.e., ±5.9% mean _V_MLSS) are also narrower compared to those of other studies predicting MLSS from LMT (±6.6% mean MLSS) (Sotero et al., 2009), OBLA (±10.3%) (Grossl et al., 2012) or other BL_R_Ts (±9.5–16.5%) (Grossl et al., 2012). The strength of the relationship and prediction accuracy reported in the current study support LE_min_ to provide a better MLSS estimation than other BL_R_Ts. This suggests _V_LE_min_, an objective submaximal variable calculated during a SD-IRT, to be one of the best single _V_MLSS predictor in endurance trained runners.

The Minimum Lactate Equivalent (LE_min_) concept was first described in the 1980s by German authors (Berg et al., 1980, 1990; Lehmann et al., 1983) and was suggested to objectively represent one of the two mentioned gold standard BLC thresholds, the exercise intensity level associated with the beginning of BLC accumulation above resting values during graded exercise, nowadays known as Lactate Threshold (LT). LE_min_ was defined as the workload corresponding to the nadir on the quadratic relationship between BLC/workload (or ${\dot{V}O}_{2}$) ratio vs. workload (or ${\dot{V}O}_{2}$) plot-data derived from an SD-IRT. Plotting BLC/workload vs. workload turns the BLC-shape during incremental exercise into a clear “U”-BLC-shape allowing the observation of BLC/workload decrement to a nadir (LE_min_) just before a clear BLC/workload increment (Figure 1). In the present study average _V_LE_min_ (11.6 km·h^−1^) approximate average _V_LT1 (11.7 km·h^−1^) (Table 1). This suggests LE_min_ to represent the pivotal equilibrium point between blood lactate production and removal (Lehmann et al., 1983; Aunola and Rusko, 1988; Berg et al., 1990). LE_min_ might be associated with several physiological characteristics and mechanisms, such as glycolytic acceleration, muscle oxidative capacity, type II muscle fiber recruitment, intramuscular lactate production, lactate release and clearance, capillary density and increasing concentrations of circulating hormones (Ivy et al., 1980; Lehmann et al., 1983; Gladden, 2004). The reason why LE_min_ would offer significant prediction advantages over other BL_R_Ts to estimate _V_MLSS can be related to: (1) the resolution of LE_min_ determination is finer than other BL_R_Ts (e.g., LT) because all the data points before and after the transition are used to project the LE_min_ value; (2) undesired error effects due to statistical scatter of the data points are minimized by the least squares curve-fitting procedure; (3) LE_min_ could essentially take on an infinite number of values, whereas LT_1_ and LT_0.2mM_ could only be based on the discrete values of the specific velocity-rate stages; (4) the troublesome identification of the first BLC elevation above baseline values (LT) due to initial BLC fluctuations associated with the error of the analyzer (Weltman et al., 1987) is resolved by the “U”-BLC-shape of LE_min_ identification without the need of a previous high level of exertion phase to induce hyperlactatemia, as it is required for LTM identification; and (5) relative changes in BLC based on the shape and slope of the BLC/workload vs. workload curve (i.e., LE_min_) during incremental exercise may be more advantageous, sensitive and robust compared with the use of absolute BLC values (i.e., FBLC thresholds) (Dickhuth et al., 1999). The relevance of LE_min_ as a predictor variable is underpinned by the fact that the other two LE_min_-related thresholds (LE_min+1mM_ and LE_min+1.5mM_) were the second and third variables best correlated with _V_MLSS. Additionally, LE_min_, LE_min+1mM_ and LE_min+1.5mM_ were the best ${\dot{V}O}_{2max}\;$ predictors, whereas average _V_LE_min+1mM_ (15.1 km·h^−1^) was nearly identical to average _V_MLSS (15.0 km·h^−1^). This suggests that _V_LE_min+1mM_ may provide a close approximation of _V_MLSS_._ These results, therefore, support LE/running-velocity to be a very good predictor of the individual and group average _V_MLSS in endurance trained runners.

A substantial relationship (*r* = 0.85) was observed between _V_MLSS and %PTV at _V_MLSS. A similar correlation magnitude (*r* = 0.83) was observed between _V_LE_min_ and %PTV at _V_LE_min_. According to our previous observations (Garcia-Tabar et al., 2015b; Llodio et al., 2015, 2016) and others (Hurley et al., 1984), these associations denote that those runners with higher _V_LE_min_ and _V_MLSS are more likely to possess their _V_LE_min_ and _V_MLSS at a higher %PTV (or %${\dot{V}O}_{2max}$) compared to those runners with lower aerobic conditioning. It also indicates that %PTV and %${\dot{V}O}_{2max}$ do not adequately differentiate across subjects, and subsequently, that the relative PTV/${\dot{V}O}_{2max}$ concept for training prescription purposes should be used with cautious (Garcia-Tabar et al., 2017). Prescribed training by relative PTV/${\dot{V}O}_{2max}$ induces different training adaptation responses (Buchheit et al., 2010), most probably due to the differed level of metabolic acidosis across individuals at a given %PTV or %${\dot{V}O}_{2max}$ (Katch et al., 1978; Meyer et al., 1999), as Figure 5 depicts. One interesting additional finding was that _V_LE_min_ to _V_MLSS ratio was remarkably homogeneous among subjects (77% _V_MLSS, range: 74–80%) in comparison with the rest of the BL_R_Ts exposed in Table 1 (e.g., 64–88% and 73–94% for LT_1_ and LT_0.2mM_, respectively). This low range (±3%) of the percentage of _V_MLSS at which _v_LE_min_ occurs is very close to the limit of the test-retest variability of MLSS workload determination (Hauser et al., 2013). This indicates that the _V_LE_min_ to _V_MLSS ratio is independent of the endurance capacity level of the assessed runners. Although _V_MLSS is substantially higher than _V_LE_min_, it is likely that some degree of commonality exists among these two physiological parameters suggesting _V_LE_min_ as a major _V_MLSS determinant. Our study advocates factors controlling _V_LE_min_ to be shared, at least partly, with those controlling _V_MLSS.

Concerning our secondary purpose, V90 was the best bloodless predictor of _V_MLSS, accounting for 64% of the variance, followed by PTV (58%) and ${\dot{V}O}_{2max}$ (36%) (Table 3). The magnitude of the relationship between V90 and _V_MLSS was similar to that between FBLC thresholds and _V_MLSS. In addition, V90 was a strong (*r* = 0.85–0.89) predictor of FBLC thresholds. These findings are in close agreement with previous HR-based studies in professional team-sport players (Garcia-Tabar et al., 2015b), elite Basque-ball players (Garcia-Tabar et al., 2017) and low-level (_V_MLSS ≈13.6 km·h^−1^) endurance runners (Kuphal et al., 2004) in which V90 was largely associated with _V_MLSS (Kuphal et al., 2004) and FBLC thresholds (Garcia-Tabar et al., 2015b, 2017). The relevance of V90 as a bloodless predictor of BL_R_Ts is strengthened by (1) the relationship between V90 and BL_R_Ts is quite stable despite alterations in BL_R_Ts due to training, detraining or hypoxia (Hurley et al., 1984; Foster et al., 1999; Friedmann et al., 2004); (2) increases in V90 have been verified to predict longitudinal training-induced improvements in FBLC thresholds (Garcia-Tabar et al., 2017); and (3) V90 is determinable during a submaximal test, i.e., maximal exertion is not always necessary (Garcia-Tabar et al., 2017), what makes V90 sometimes more suitable than PTV and ${\dot{V}O}_{2max}$. Results indicate V90 to be an appealing variable since it is a valid, easy, noninvasive and low-cost suitable estimator of _V_MLSS and FBLC thresholds during a progressive running test in endurance trained runners facilitating the monitoring of aerobic conditioning.

During exercise at _V_MLSS, absolute HR markedly differed between subjects. Average relative HR (%HR_max_), instead, was maintained within a reasonably narrow range over time (85–92% from min 5 to 30), although, in agreement with other studies in runners (Haverty et al., 1988; Llodio et al., 2016), it also significantly increased over time (Figure 3). This suggests that a HR zone, rather than a fixed absolute or relative HR value, should be considered during training sessions when the goal is to reach an exercise intensity related to _V_MLSS. However, the individual %HR_max_ values during _V_MLSS CVRTs varied considerably between individuals, ranging from 81 to 85% HR_max_ and from 85 to 98% HR_max_ after 5 and 30 min of exercise, respectively. This indicates that the HR zone corresponding to MLSS should be estimated on individual basis (Llodio et al., 2016). An interesting finding was the extremely large relationship observed between the individual absolute _HR_LE_min_ and the individual absolute HR values after 5 min at _V_MLSS (Figure 6). This association suggests that _HR_LE_min_ can be accurately used to predict HR value after 5 min at _V_MLSS. Determination of _V_LE_min_ and its corresponding HR is therefore advantageous over other BL_R_Ts (e.g., LTM) whose HR values are not usable for training monitoring purposes (Messias et al., 2017, 2018).

The present study is limited in some aspects. First, the applicability of the results is limited to a homogeneous sample of relatively young male runners with _V_MLSS values ranging from 13.3 to 16.5 km·h^−1^ (i.e., _V_LE_min_ values from 10.5 to 12.6 km·h^−1^). It is possible that the results might differ for individuals with higher or lower _V_MLSS values. The same holds true for gender, because specific prediction models have not been developed for females. Second, the reported prediction equations are only recommended to be used with the specific testing procedures utilized and described in this study. It is known that BLC- and HR-based variables might be influenced by factors such as the blood sampling methods, pre-testing physical status, hydration and nutritional status, dietary or pharmacological manipulations, and environmental conditions (Halson, 2014). In addition, the choice of an appropriate initial running velocity and increment rate between stages utilized in the SD-IRT is also an essential aspect to permit fine resolution of LT and LE_min_ (Hollmann, 1985; Aunola and Rusko, 1988). The initial running velocity and the increment rate must be sufficiently small to allow enough data-points below the location of the LE_min_ to permit an adequate analysis of the two-segment model. Third, a test-retest analysis of LE_min_ was beyond the scope of this study, and therefore, whether LE_min_ is reliable was not verified. Dickhuth et al. (1999), however, found a good test-retest reproducibility (*r* = 0.90) of LE_min_ determined during a SD-IRT in young males. Finally, this study is a cross-sectional study. The almost perfect (Hopkins et al., 2009) relationship observed between _V_LE_min_ and _V_MLSS in this predictive cross-sectional study does not necessarily imply that changes observed over a period of time in _V_LE_min_ would predict changes in _V_MLSS. Further longitudinal studies are required to examine whether longitudinal training-induced changes in _V_MLSS could be predicted and monitored by _V_LE_min_, as well as to clarify the degree of commonality between these two parameters. Despite these limitations, the results of the present study provide important and novel information about the prediction of MLSS from LE_min_.

In conclusion, results of the current study indicate that when BLC assessment is available but only one testing session is feasible, _V_LE_min_ determined during a SD-IRT is a very good predictor of _V_MLSS in endurance trained runners. Average _V_LE_min_ resulted in similar mean value than the classical Lactate Threshold (LT_1_). Accuracy in MLSS prediction by LE_min_ found in this study is among the highest reported in the literature. LE_min_ is a continuous rather than a discrete variable and the minimum point on a U-shaped curve using the least squares curve-fitting procedure is determinable with a very fine resolution minimizing error effects due to statistical scatter of the data points. The current study, therefore, suggests _V_LE_min_, an objective submaximal variable, to be probably the best single _V_MLSS predictor in endurance trained runners. If direct BLC measurement is undesirable or unfeasible, V90 is a non-invasive fairly good predictor of _V_MLSS. Precise estimation of _V_MLSS from a single-session discontinuous progressive running test is a reasonable alternative to reduce costs and considerably alleviate the burden associated with the classical MLSS assessment.

## Author contributions

EG and IG-T equally contributed to the conception and design of the experiments; performing of the experiments; acquisition, registration, analysis and interpretation of the data; preparation of figures; and drafting of the manuscript; EG and IG-T warmly discussed about the manuscript; and critically reviewed and edited the drafts; EG and IG-T approved the final version of the manuscript.

### Conflict of interest statement

The authors declare that the research was conducted in the absence of any commercial or financial relationships that could be construed as a potential conflict of interest.

## References

[B1] Aunola S. Rusko H. (1988). Comparison of two methods for aerobic threshold determination. Eur. J. Appl. Physiol. Occup. Physiol. 57, 420–424. 10.1007/BF00417987 3396556

[B2] Barr D. P. Himwich H. E. (1923). Studies in the physiology of muscular exercise: III. Development and duration of changes in acid-base equilibrium. J. Biol. Chem. 55, 539–555

[B3] Beneke R. (1995). Anaerobic threshold, individual anaerobic threshold, and maximal lactate steady state in rowing. Med. Sci. Sports Exerc. 27, 863–867. 10.1249/00005768-199506000-00010 7658947

[B4] Berg A. Jakob E. Lehmann M. Dickhuth H. H. Huber G. Keul J. (1990). Current aspects of modern ergometry. Pneumologie 44, 2–13. 2408033

[B5] Berg A. Stippig J. Keul J. Huber G. (1980). Zur Beurteilung der Leistungsfähigkeit und Belastbarkeit von Patienten mit coronarer Herzkrankheit. Dtsch. Z. Sportmed. 31, 199–205.

[B6] Brooks G. A. (1985). Anaerobic threshold: review of the concept and directions for future research. Med. Sci. Sports Exerc. 17, 22–34. 10.1249/00005768-198502000-00005 3884959

[B7] Buchheit M. Chivot A. Parouty J. Mercier D. Al Haddad H Laursen P. B.. (2010). Monitoring endurance running performance using cardiac parasympathetic function. Eur. J. Appl. Physiol. 108, 1153–1167. 10.1007/s00421-009-1317-x 20033207

[B8] Cheng B. Kuipers H. Snyder A. C. Keizer H. A. Jeukendrup A. Hesselink M. (1992). A new approach for the determination of ventilatory and lactate thresholds. Int. J. Sports Med. 13, 518–522. 10.1055/s-2007-1021309 1459746

[B9] Coyle E. F. Coggan A. R. Hopper M. K. Walters T. J. (1988). Determinants of endurance in well-trained cyclists. J. Appl. Physiol. (1985) 64, 2622–2630. 10.1152/jappl.1988.64.6.2622 3403447

[B10] Denadai B. S. Gomide E. B. Greco C. C. (2005). The relationship between onset of blood lactate accumulation, critical velocity, and maximal lactate steady state in soccer players. J. Strength Cond. Res. 19, 364–368. 10.1519/1533-4287(2005)19[364:TRBOOB]2.0.CO;2 15903376

[B11] Dickhuth H. H. Yin L. Niess A. Röcker K. Mayer F. Heitkamp H. C.. (1999). Ventilatory, lactate-derived and catecholamine thresholds during incremental treadmill running: relationship and reproducibility. Int. J. Sports Med. 20, 122–127. 10.1055/s-2007-971105 10190774

[B12] Farrell P. A. Wilmore J. H. Coyle E. F. Billing J. E. Costill D. L. (1979). Plasma lactate accumulation and distance running performance. Med. Sci. Sports 11, 338–344. 10.1249/00005768-197901140-00005 530025

[B13] Faude O. Kindermann W. Meyer T. (2009). Lactate threshold concepts: how valid are they? Sports Med. 39, 469–490. 10.2165/00007256-200939060-00003 19453206

[B14] Ferguson B. S. Rogatzki M. J. Goodwin M. L. Kane D. A. Rightmire Z. Gladden L. B. (2018). Lactate metabolism: historical context, prior misinterpretations, and current understanding. Eur. J. Appl. Physiol. 118, 691–728. 10.1007/s00421-017-3795-6 29322250

[B15] Figueira T. R. Caputo F. Pelarigo J. G. Denadai B. S. (2008). Influence of exercise mode and maximal lactate-steady-state concentration on the validity of OBLA to predict maximal lactate-steady-state in active individuals. J. Sci. Med. Sport 11, 280–286. 10.1016/j.jsams.2007.02.016 17553745

[B16] Foster C. Fitzgerald D. J. Spatz P. (1999). Stability of the blood lactate-heart rate relationship in competitive athletes. Med. Sci. Sports Exerc. 31, 578–582. 10.1097/00005768-199904000-00014 10211855

[B17] Friedmann B. Bauer T. Menold E. Bärtsch P. (2004). Exercise with the intensity of the individual anaerobic threshold in acute hypoxia. Med. Sci. Sports Exerc. 36, 1737–1742. 10.1249/01.MSS.0000142307.62181.37 15595295

[B18] Garcia-Tabar I. Eclache J. P. Aramendi J. F. Gorostiaga E. M. (2015a). Gas analyzer's drift leads to systematic error in maximal oxygen uptake and maximal respiratory exchange ratio determination. Front. Physiol. 6:308. 10.3389/fphys.2015.00308 26578980 PMC4626835

[B19] Garcia-Tabar I. Izquierdo M. Gorostiaga E. M. (2017). On-field prediction vs monitoring of aerobic capacity markers using submaximal lactate and heart rate measures. Scand. J. Med. Sci. Sports 27, 462–473. 10.1111/sms.12853 28181710

[B20] Garcia-Tabar I. Llodio I. Sánchez-Medina L. Ruesta M. Ibañez J. Gorostiaga E. M. (2015b). Heart rate-based prediction of fixed blood lactate thresholds in professional team-sport players. J. Strength Cond. Res. 29, 2794–2801. 10.1519/JSC.0000000000000957 25844867

[B21] Gladden L. B. (2004). Lactate metabolism: a new paradigm for the third millennium. J. Physiol. 558, 5–30. 10.1113/jphysiol.2003.058701 15131240 PMC1664920

[B22] Grossl T. De Lucas R. D. De Souza K. M. Antonacci Guglielmo L. G. (2012). Maximal lactate steady-state and anaerobic thresholds from different methods in cyclists. Eur. J. Sport Sci. 12, 161–167. 10.1080/17461391.2010.551417

[B23] Halson S. L. (2014). Monitoring training load to understand fatigue in athletes. Sports Med. 44, S139–S147. 10.1007/s40279-014-0253-z 25200666 PMC4213373

[B24] Hauser T. Bartsch D. Baumgärtel L. Schulz H. (2013). Reliability of maximal lactate-steady-state. Int. J. Sports Med. 34, 196–199. 10.1055/s-0032-1321719 22972242

[B25] Haverty M. Kenney W. L. Hodgson J. L. (1988). Lactate and gas exchange responses to incremental and steady state running. Br. J. Sports Med. 22, 51–54. 10.1136/bjsm.22.2.51 3167501 PMC1478544

[B26] Heck H. Mader A. Hess G. Mücke S. Müller R. Hollmann W. (1985). Justification of the 4-mmol/l lactate threshold. Int. J. Sports Med. 6, 117–130. 10.1055/s-2008-1025824 4030186

[B27] Hedges L. V. (1981). Distribution theory for Glass's estimator of effect size and related estimators. J. Educ. Behav. Stat. 6, 107–128 10.3102/10769986006002107

[B28] Hollmann W. (1985). Historical remarks on the development of the aerobic-anaerobic threshold up to 1966. Int. J. Sports Med. 6, 109–116. 10.1055/s-2008-1025823 3897079

[B29] Hopkins W. G. Marshall S. W. Batterham A. M. Hanin J. (2009). Progressive statistics for studies in sports medicine and exercise science. Med. Sci. Sports Exerc. 41, 3–13. 10.1249/MSS.0b013e31818cb278 19092709

[B30] Hurley B. F. Hagberg J. M. Allen W. K. Seals D. R. Young J. C. Cuddihee R. W.. (1984). Effect of training on blood lactate levels during submaximal exercise. J. Appl. Physiol. Respir. Environ. Exerc. Physiol. 56, 1260–1264. 10.1152/jappl.1984.56.5.1260 6725086

[B31] Ivy J. L. Withers R. T. Van Handel P. J. Elger D. H. Costill D. L. (1980). Muscle respiratory capacity and fiber type as determinants of the lactate threshold. J. Appl. Physiol. Respir. Environ. Exerc. Physiol. 48, 523–527. 10.1152/jappl.1980.48.3.523 7372524

[B32] Jones A. M. Doust J. H. (1998). The validity of the lactate minimum test for determination of the maximal lactate steady state. Med. Sci. Sports Exerc. 30, 1304–1313. 10.1097/00005768-199808000-00020 9710874

[B33] Jones N. L. Ehrsam R. E. (1982). The anaerobic threshold. Exerc. Sport Sci. Rev. 10, 49–83. 10.1249/00003677-198201000-00003 6811284

[B34] Katch V. Weltman A. Sady S. Freedson P. (1978). Validity of the relative percent concept for equating training intensity. Eur. J. Appl. Physiol. Occup. Physiol. 39, 219–227. 10.1007/BF00421445 710387

[B35] Kindermann W. Simon G. Keul J. (1978). Dauertraining–Ermittlung der optimalen Trainingsherzfrequenz und Leistungsfähigkeit. Leistungssport 8, 34–39.

[B36] Krouwer J. S. (2008). Why Bland-Altman plots should use X, not (Y+X)/2 when X is a reference method. Stat. Med. 27, 778–780. 10.1002/sim.3086 17907247

[B37] Kuphal K. E. Potteiger J. A. Frey B. B. Hise M. P. (2004). Validation of a single-day maximal lactate steady state assessment protocol. J. Sports Med. Phys. Fitness 44, 132–140. 10.1097/00005768-200105001-01374 15470310

[B38] Lehmann M. Berg A. Kapp R. Wessinghage T. Keul J. (1983). Correlations between laboratory testing and distance running performance in marathoners of similar performance ability. Int. J. Sports Med. 4, 226–230. 10.1055/s-2008-1026039 6654546

[B39] Llodio I. Garcia-Tabar I. Sánchez-Medina L. Ibanez J. Gorostiaga E. M. (2015). Estimation of the maximal lactate steady state in junior soccer players. Int. J. Sports Med. 36, 1142–1148. 10.1055/s-0035-1554643 26332904

[B40] Llodio I. Gorostiaga E. M. Garcia-Tabar I. Granados C. Sánchez-Medina L. (2016). Estimation of the maximal lactate steady state in endurance runners. Int. J. Sports Med. 37, 539–546. 10.1055/s-0042-102653 27116348

[B41] Messias L. H. D. Gobatto C. A. Beck W. R. Manchado-Gobatto F. B. (2017). The lactate minimum test: concept, methodological aspects and insights for future investigations in human and animal models. Front. Physiol. 8:389. 10.3389/fphys.2017.00389 28642717 PMC5463055

[B42] Messias L. H. D. Polisel E. E. C. Manchado-Gobatto F. B. (2018). Advances of the reverse lactate threshold test: non-invasive proposal based on heart rate and effect of previous cycling experience. PLoS ONE 13:e0194313. 10.1371/journal.pone.0194313 29534108 PMC5849329

[B43] Meyer T. Gabriel H. H. Kindermann W. (1999). Is determination of exercise intensities as percentages of VO_2max_ or HR_max_ adequate? Med. Sci. Sports Exerc. 31, 1342–1345. 10.1097/00005768-199909000-00017 10487378

[B44] Mezzani A. Hamm L. F. Jones A. M. McBride P. E. Moholdt T. Stone J. A.. (2012). Aerobic exercise intensity assessment and prescription in cardiac rehabilitation: a joint position statement of the European Association for Cardiovascular Prevention and Rehabilitation, the American Association of Cardiovascular and Pulmonary Rehabilitation, and the Canadian Association of Cardiac Rehabilitation. J. Cardiopulm. Rehabil. Prev. 32, 327–350. 10.1097/HCR.0b013e3182757050 23103476

[B45] Owles W. H. (1930). Alterations in the lactic acid content of the blood as a result of light exercise, and associated changes in the co(2)-combining power of the blood and in the alveolar co(2) pressure. J. Physiol. 69, 214–237. 10.1113/jphysiol.1930.sp002646 16994099 PMC1402944

[B46] Philp A. Macdonald A. L. Carter H. Watt P. W. Pringle J. S. (2008). Maximal lactate steady state as a training stimulus. Int. J. Sports Med. 29, 475–479. 10.1055/s-2007-965320 18302077

[B47] Seiler S. (2010). What is best practice for training intensity and duration distribution in endurance athletes? Int. J. Sports Physiol. Perform. 5, 276–291. 10.1123/ijspp.5.3.276 20861519

[B48] Sotero R. C. Pardono E. Landwehr R. Campbell C. S. Simoes H. G. (2009). Blood glucose minimum predicts maximal lactate steady state on running. Int. J. Sports Med. 30, 643–646. 10.1055/s-0029-1220729 19569005

[B49] Stratton E. O'Brien B. J. Harvey J. Blitvich J. McNicol A. J. Janissen D.. (2009). Treadmill velocity best predicts 5000-m run performance. Int. J. Sports Med. 30, 40–45. 10.1055/s-2008-1038761 19202577

[B50] Tegtbur U. Busse M. W. Braumann K. M. (1993). Estimation of an individual equilibrium between lactate production and catabolism during exercise. Med. Sci. Sports Exerc. 25, 620–627. 10.1249/00005768-199305000-00015 8492691

[B51] Van Schuylenbergh R. Vanden Eynde B. Hespel P. (2004). Correlations between lactate and ventilatory thresholds and the maximal lactate steady state in elite cyclists. Int. J. Sports Med. 25, 403–408. 10.1055/s-2004-819942 15346226

[B52] Vobejda C. Fromme K. Samson W. Zimmermann E. (2006). Maximal constant heart rate–a heart rate based method to estimate maximal lactate steady state in running. Int. J. Sports Med. 27, 368–372. 10.1055/s-2005-865717 16729378

[B53] Wasserman K. Whipp B. J. Koyl S. N. Beaver W. L. (1973). Anaerobic threshold and respiratory gas exchange during exercise. J. Appl. Physiol. 35, 236–243. 10.1152/jappl.1973.35.2.236 4723033

[B54] Weltman A. Snead D. Seip R. Schurrer R. Levine S. Rutt R.. (1987). Prediction of lactate threshold and fixed blood lactate concentrations from 3200-m running performance in male runners. Int. J. Sports Med. 8, 401–406. 10.1055/s-2008-1025694 3429086

[B55] Weltman A. Snead D. Stein P. Seip R. Schurrer R. Rutt R.. (1990). Reliability and validity of a continuous incremental treadmill protocol for the determination of lactate threshold, fixed blood lactate concentrations, and VO_2max_. Int. J. Sports Med. 11, 26–32. 10.1055/s-2007-1024757 2318561

[B56] Yeh M. P. Gardner R. M. Adams T. D. Yanowitz F. G. Crapo R. O. (1983). “Anaerobic threshold”: problems of determination and validation. J. Appl. Physiol. Respir. Environ. Exerc. Physiol. 55, 1178–1186. 10.1152/jappl.1983.55.4.1178 6629951

[B57] Yoshida T. (1984). Effect of exercise duration during incremental exercise on the determination of anaerobic threshold and the onset of blood lactate accumulation. Eur. J. Appl. Physiol. Occup. Physiol. 53, 196–199. 10.1007/BF00776589 6542852

[B58] Yoshida T. Udo M. Iwai K. Chida M. Ichioka M. Nakadomo F.. (1990). Significance of the contribution of aerobic and anaerobic components to several distance running performances in female athletes. Eur. J. Appl. Physiol. Occup. Physiol. 60, 249–253. 10.1007/BF00379391 2357979

